# Volatile organic compounds emitted by *Burkholderia pyrrocinia* CNUC9 trigger induced systemic salt tolerance in *Arabidopsis thaliana*

**DOI:** 10.3389/fmicb.2022.1050901

**Published:** 2022-11-17

**Authors:** Huan Luo, Myoungjoo Riu, Choong-Min Ryu, Jun Myoung Yu

**Affiliations:** ^1^Department of Applied Biology, Chungnam National University, Daejeon, South Korea; ^2^Molecular Phytobacteriology Laboratory, Infectious Disease Research Center, KRIBB, Daejeon, South Korea

**Keywords:** plant growth-promoting rhizobacteria, *Burkholderia pyrrocinia*, volatile compounds, plant growth promotion, induced systemic salt tolerance

## Abstract

Salinity is among the most significant abiotic stresses that negatively affects plant growth and agricultural productivity worldwide. One ecofriendly tool for broadly improving plant tolerance to salt stress is the use of bio-inoculum with plant growth-promoting rhizobacteria (PGPR). In this study, a bacterium strain CNUC9, which was isolated from maize rhizosphere, showed several plant growth-promoting characteristics including the production of 1-aminocyclopropane-1-carboxylate deaminase, indole acetic acid, siderophore, and phosphate solubilization. Based on 16S rRNA and *recA* gene sequence analysis, we identified strain CNUC9 as *Burkholderia pyrrocinia*. Out of bacterial determinants to elicit plant physiological changes, we investigated the effects of volatile organic compounds (VOCs) produced by *B*. *pyrrocinia* CNUC9 on growth promotion and salinity tolerance in *Arabidopsis thaliana*. Higher germination and survival rates were observed after CNUC9 VOCs exposure under 100 mM NaCl stress. CNUC9 VOCs altered the root system architecture and total leaf area of *A*. *thaliana* compared to the control. *A. thaliana* exposed to VOCs induced salt tolerance by increasing its total soluble sugar and chlorophyll content. In addition, lower levels of reactive oxygen species, proline, and malondialdehyde were detected in CNUC9 VOCs-treated *A*. *thaliana* seedlings under stress conditions, indicating that VOCs emitted by CNUC9 protected the plant from oxidative damage induced by salt stress. VOC profiles were obtained through solid-phase microextraction and analyzed by gas chromatography coupled with mass spectrometry. Dimethyl disulfide (DMDS), methyl thioacetate, and 2-undecanone were identified as products of CNUC9. Our results indicate that optimal concentrations of DMDS and 2-undecanone promoted growth in *A*. *thaliana* seedlings. Our findings provide greater insight into the salt stress alleviation of VOCs produced by *B*. *pyrrocinia* CNUC9, as well as potential sustainable agriculture applications.

## Introduction

Soil salinity is one of the most significant environmental constraints restricting seed germination, plant growth, and productivity worldwide, posing a serious threat to global food security (Mukhopadhyay et al., 2021). Globally, approximately 20% of agricultural areas and 33% of irrigated lands are negatively affected by soil salinity, and the salinization of irrigated land is predicted to increase by 50% by 2050 (Shrivastava and Kumar, 2015). Salinity adversely affects plant physiology, biochemistry, and metabolism that induces cellular osmotic imbalance, inhibited root growth, and alters root architecture, impairing the ability of plants to acquire water and nutrients (Nawaz et al., 2020). High salinity also induces the accumulation of reactive oxygen species (ROS), which are detrimental to cell viability, photosynthetic pigments, membrane lipid integrity, and phytohormone imbalance (Cappellari et al., 2020; Ali et al., 2022). Various strategies have been developed to mitigate the effects of soil salinity on crops, including the reclamation of saline soil and breeding salt-tolerant plant varieties through genetic engineering (Nawaz et al., 2020). However, these methods have not been widely accepted due to time and resource costs, as well as potential environmental risks (Hu et al., 2012). Therefore, the application of microorganisms that are beneficial to plants [e.g., plant growth-promoting rhizobacteria (PGPR)] has gained attention as an alternative sustainable agricultural approach to alleviating salt stress in crops.

PGPR are plant root-associated bacteria that improve plant growth and increase tolerance to various abiotic and biotic stresses (Ha Tran et al., 2021). The main mechanisms of growth and yield improvement are the production of growth regulators such as indole acetic acid (IAA), gibberellic acid, and cytokinins; processes increasing nutrient availability such as nitrogen fixation, phosphorous solubilization, and siderophore production; synthesis of 1-amino cyclopropane-1-carboxylic acid (ACC) deaminase; and secretion of exopolysaccharides (EPS) and antimicrobial secondary metabolites (Bhattacharyya and Jha, 2012; Gouda et al., 2018; Basu et al., 2021). In addition, volatile organic compounds (VOCs) produced by PGPR have been shown to enhance plant biomass, disease resistance, and abiotic stress tolerance (Farag et al., 2013; Fincheira and Quiroz, 2018; Fincheira et al., 2021).

VOCs emitted by plant-associated microorganisms are low-molecular-weight (<300 g mol^−1^) lipophilic compounds with a low boiling point and high vapor pressure. These characteristics allow them to act as signals *via* short-and long-distance dispersal in the rhizosphere, allowing indirect interactions between plants and microorganisms (Cordovez et al., 2018). Since the production of VOCs by rhizobacteria was first reported to trigger growth promotion in *A*. *thaliana* (Ryu et al., 2003), several studies have demonstrated that VOCs released from rhizobacteria such as *Bacillus*, *Burkholderia*, *Enterobacter*, *Pseudomonas*, *Streptomyces*, and *Stenotrophomonas* species can stimulate plant growth and induced systemic tolerance (IST) against abiotic stresses (Yang et al., 2009; Fincheira and Quiroz, 2018; Veselova et al., 2019; Cellini et al., 2021). Some studies reported that bacterial VOCs promote plant growth by influencing root architecture and growth, resulting in increased surface area for nutrient and water acquisition as well as other rhizosphere effects (Gutiérrez-Luna et al., 2010; Grover et al., 2021). The number of chloroplasts and chlorophyll content increase following exposure to *Bacillus subtilis* GB03 VOCs to *A*. *thaliana*, resulting in higher quantum efficiency, complex II photosynthetic efficiency, and effective quantum yield (Zhang et al., 2008). VOCs also regulate growth hormone redistribution, induce key defense mechanisms, and increase antioxidant enzyme accumulation (Kwon et al., 2010).

The *Burkholderia cepacia* complex (Bcc) is the primary cluster in the *Burkholderia* genus, and is found in diverse environmental niches (Meier-Kolthoff et al., 2022). Some Bcc species have been used as agricultural biocontrol agents or in toxin bioremediation (LiPuma and Mahenthiralingam, 1999). Bibi et al. (2022) reported that the application of *B*. *cenocepacia* as a bio-organic fertilizer increased the maize germination index, promptness index, and seedling vigor index by 32, 34, and 21%, respectively, compared to controls. *B. phytofirmans* strain PsJN enhances *A*. *thaliana* growth and salt tolerance throughout its life cycle, modifying ion transporters necessary for salt-stress tolerance (Pinedo et al., 2015). Sarkar et al. (2018) reported that *Burkholderia* sp. MTCC 12259 was tolerant to treatment with 1.2 M NaCl and produced IAA, EPS, and proline essential for rice seedling growth under salt stress. Subsequent studies have mainly focused on the antagonistic effects of *B. pyrrocinia* (Vandamme et al., 2002; Ren et al., 2011; de los Santos-Villalobos et al., 2012; Depoorter et al., 2016). However, the mechanisms of its VOCs-mediated growth promotion and IST in plants against abiotic stresses, particularly salt stress are currently limited.

In this study, we screened 24 bacterial isolates from maize rhizosphere, and selected one isolate that identified as *B*. *pyrrocinia* (strain CNUC9) has various plant growth promoting traits. Considering the potential role of bacterial VOCs in growth promotion and plant stress tolerance, we investigated the role of CNUC9 VOCs on plant biomass (vigor and morphological characteristics of leaf and root), and the mitigation of salt stress in *A. thaliana.* In addition, we evaluated stress-related biochemical changes in cellular solutes and ROS generation in *A. thaliana* under salt stress.

## Materials and methods

### Sample collection and rhizobacterium isolation

Rhizosphere soil samples were collected from roots of maize (*Zea mays* L.) grown in Gongju, South Korea (36°21′34.6”N 127°09′48.4″E), in 2020. Roots and adhered soil were manually separated from the surrounding bulk soil and collected into sterile polyethylene bags. To isolate rhizobacteria, roots were gently washed with tap water, and 5 g root tips were transferred to 50 ml 0.8% NaCl (w/v) solution. Bacteria were removed from root tips by vortexing and sonication three times each for 30 s. The suspension was serially diluted and each dilution was separately spread on nutrient agar (NA) plates. After 3 days of incubation at 28°C, bacterial colonies with different phenotypes were purified. Purified bacterial isolates were stored in 40% glycerol at −80°C and used for further study.

### Screening of bacterial isolates for plant growth-promoting traits

To screen for phosphate solubilization, 10 μl each bacterial isolate was inoculated onto Pikovaskya’s agar medium containing tri-calcium phosphate as the mineral P (Pikovskaya, 1948) and incubated at 28°C for 4 days. Halo zone formation by the colonies was considered to indicate phosphate-solubilizing capacity. IAA production by bacterial strains was estimated in LB broth supplemented with L-tryptophan (100 mg/l) using the Salkowski reagent, which consisted of 0.5 M of FeCl_3_ in 70% HClO_4_ (Bric et al., 1991). The IAA concentration was determined from a standard curve of purified IAA (Daejung Chemicals and Metals Co. Ltd., Siheung, Korea). To screen for siderophore production, 10 μl each bacterial isolate was inoculated onto Chrome Azurol Sulphonate over-laid onto NA medium (Alexander and Zuberer, 1991). A color change observed on the overlaid medium after 3 days was considered to indicate siderophore production. Proteolytic activity was determined by streaking isolates on skim milk agar, and the formation of clear zones surrounding the colonies was considered to indicate casein hydrolyzation and the formation of soluble nitrogenous compounds. ACC deaminase production was screened by culturing bacteria in Dwarkin and Foster (DF) medium using ACC (Thermo Fisher Scientific, Waltham, MA, United States) as the sole nitrogen source (Dworkin and Foster, 1958). Quantification of ACC deaminase activity was performed spectrophotometrically by measuring the production of α-ketobutyrate at 540 nm by comparing it with the standard curve of different concentrations of purified α-ketobutyrate (Sigma, United States; Penrose and Glick, 2003).

### Molecular identification of rhizobacteria

For taxonomical identification of the bacterial isolates, 16S rRNA and *recA* genes were amplified and sequenced using the primers 27 F and 1492 R (Pitcher et al., 1989), and BCR1 and BCR2 (Mahenthiralingam et al., 2000), respectively. Multiple sequence alignments were generated with the 16S rRNA and *recA* sequences of strain CNUC9, and available sequences of related species were downloaded from the National Center for Biotechnology Information (NCBI) databank. A phylogenetic tree was constructed using the MEGA v7.0 software (Kumar et al., 2018) based on maximum likelihood (ML) analysis. The best-fit model of molecular evolution with 1,000 bootstrap replicates was computed, and bootstrap values >80% were considered highly supported.

### Screening of water-and salt-stress tolerant strains

To screen drought stress-tolerant bacteria, isolates (20 μl) were grown in 20 ml LB broth containing 15 and 30% PEG 6000 at 28°C with rapid shaking. We also screened for salinity tolerance by growing bacterial strains in 20 ml LB broth with different salt concentrations (0, 400, 600, 800, or 1,000 mM NaCl). Samples were collected periodically and optical density at 620 nm (OD_620_) was measured using a spectrophotometer. A bacterial OD_620_ ≥ 0.1 was considered to indicate tolerance. Distilled water (DW)-inoculated LB medium was used as a control.

### Plant materials and growth conditions

*Arabidopsis thaliana* Col-0 seeds were surface-sterilized with 70% ethyl alcohol for 2 min and 1% sodium hypochlorite solution for 1 min, and then rinsed five times with sterile water. Five *A*. *thaliana* seeds were sown in one half of a two-section I-plate containing 1/2-strength Murashige and Skoog medium (1/2 MS) with 1% agar, and a 20 μl bacterial aliquot was spotted onto the other half of the I-plate containing NA medium. Four treatment combinations were prepared as follows: DW inoculation/0 mM NaCl, CNUC9 inoculation/0 mM NaCl, DW inoculation/100 mM NaCl, and CNUC9 inoculation/100 mM NaCl. Plates were sealed with parafilm and placed vertically in a growth chamber at 22°C with a 16 h/8 h light/dark photoperiod. After 10 days, plant growth parameters were recorded.

### Seedling physiological traits

To determine seed germination and seedling survival rates, sterilized *A*. *thaliana* seeds were sown as described above. A total of 200 seeds for each treatment were used, each with three replicates. Root lengths >0.5 cm were considered to indicate survival. The germination and survival rates of different treatments were counted 10 days after sowing.

To analyze root architecture and leaf proliferation, *A*. *thaliana* seedlings in Petri dishes were directly scanned using a scanner (Perfection V850 Pro, Epson, Nagano, Japan), and scanned images were analyzed using the WinRHIZO image analysis system for *A*. *thaliana* (Regent Instruments, Inc., Quebec City, QC, Canada). Link and color analyses were used to detect total root length, root surface area, lateral root number, and leaf area. Microscopic differences in root architectures under salt stress among treatments were observed by microscopy (BX41, Olympus, Tokyo, Japan).

### Plant biochemical analyses

Chlorophyll a (Chl a) and b (Chl b) and total chlorophyll content were determined by spectrophotometric analysis as described previously (Inskeep and Bloom, 1985). Briefly, 0.1 g seedling tissues were ground with liquid nitrogen, then transferred to a tube containing 1 ml 80% acetone. The mixtures were vortexed to homogenize the leaf tissues and centrifuged at 13,000 ×*g* for 10 min at 4°C. The supernatant was measured at OD of 663.6 and 646.6 nm. Chl a, Chl b, and total chlorophyll concentrations were calculated as follows (Porra, 2002):

[Chl a] = 13.71 × A_663.6_–2.85 × A_646.6_,

[Chl b] = 22.39 × A_646.6_–5.42 × A_663.6_,

[Total chlorophyll] = [Chl a] + [Chl b],

where A is absorbance at the indicated wavelength.

To determine the total sugar content, seedlings were ground using liquid nitrogen, and 0.1 g powder was homogenized with 10 ml sterile distilled water. Samples were vortexed and boiled for 1 h. To remove chlorophyll, 0.1 g activated charcoal was added and the mixture was boiled again for 30 min. Homogenized samples were centrifuged at 13,000 ×*g* for 10 min. Then, 200 μl supernatant was transferred to a new tube and 1 ml 0.2% anthrone was added. After boiling again for 30 min, the samples were transferred in an ice bath to stop the reaction. We recorded OD_620_ and standard curves were drawn for different sucrose concentrations as described previously (Ikram et al., 2018).

The proline content of *A*. *thaliana* seedlings was quantified as described previously (Bates et al., 1973). Briefly, 0.1 g ground tissues were homogenized with 1 ml 3% aqueous sulfosalicylic acid by vortexing, and then the samples were centrifuged at 13,000 ×*g* for 10 min. The 200 μl supernatant was mixed with 500 μl glacial acetic acid and 500 μl acidic ninhydrin. After boiling for 30 min, samples were transferred to an ice bath to stop the reaction. OD_520_ was recorded, and different concentrations of L-proline were used as standards.

Lipid peroxidation in *A*. *thaliana* seedlings was estimated to be malondialdehyde (MDA) content by calculating the amount of MDA extracted from 0.5% (w/v) thiobarbituric acid and 1% (w/v) trichloroacetic acid as described previously (Du and Bramlage, 1992). The OD of the supernatant was measured at 450, 532, and 600 nm by spectrophotometry, and MDA concentrations (μmol/g) were calculated as follows: [MDA] = 6.45 (A_532_–A_600_)–0.56 A_450_, where A is absorbance at the indicated wavelength.

Hydrogen peroxide (H_2_O_2_) content was detected by 3,3′-diaminobenzidine (DAB) staining as described previously (Asselbergh et al., 2007), and quantified as described previously (Mukherjee and Choudhuri, 1983). Different concentrations of H_2_O_2_ obtained from Sigma-Aldrich (St. Louis, MO, United States) were used as standards.

### VOC analysis by solid-phase microextraction with gas chromatography–mass spectrometry (SPME–GC–MS)

Bacterial VOCs were collected using a solid-phase microextraction (SPME) fiber with 50/30 μm divinyl benzene/carboxen/polydimethylsiloxane (Supelco, Bellefonte, PA, United States) and an autosampler (CombiPAL, CTC Analytics, Zwingen, Switzerland). For VOCs sample preparation, a bacterial aliquot (20 μl) was inoculated into NA medium and the plates were incubated under the same conditions described for the plant growth assay. Fibers were introduced and held for 15 min in the bottle’s headspace at 50°C. For GC–MS analysis, we employed an Agilent 7890A series gas chromatograph (Agilent Technologies, Santa Clara, CA, United States) equipped with an HP-5MS capillary column (30 m length, 0.25 mm inner diameter, 0.25 μm film thickness) coupled with a Triple-Axis Detector (Agilent Technologies). The equipment was operated under the following conditions: automatic sample desorption with the injector port at 250°C, oven programmed with an initial temperature of 40°C to be held for 3 min, and then increased at a rate of 10°C min^−1^ to 220°C. Helium was used as the carrier gas (flow rate: 1.0 ml/min). Electron impact ionization at 200°C was conducted to analyze mass fragments in a scan range of 40–500 m/z. GC–MS analysis was performed independently for bacteria grown alone and for the culture media. Data analysis and compound identification were performed using the National Institute of Standards and Technology Mass Spectral Database (NIST 11.L).

### Plant growth promotion assay using selected synthetic VOCs

To determine the effect of each identified VOC, synthetic dimethyl disulfide (Sigma-Aldrich), methyl thioacetate (TCI, Tokyo, Japan), and 2-undecanone (Sigma-Aldrich) were dissolved in dimethyl sulfoxide (DMSO). Different concentrations of each compound (0.2 μM, 2 μM, and 20 μM) and controls (50 μl DMSO and DW) were applied to a sterile paper disk (diameter: 1.85 mm), and placed on one half of an I-plate. *A. thaliana* seeds were sown on the opposite side of the I-plate containing 1/2 MS medium. The sealed plates were incubated as described above. After 10 days, the effects of individual synthetic compounds on plant growth were recorded.

### Data analysis

All data presented in bar graphs are means ± standard errors of the mean (SEMs). Means were compared using analysis of variance (ANOVA), followed by Tukey’s *post hoc* test for multiple comparisons. All tests were performed using the GraphPad Prism 9 (GraphPad Software, San Diego, CA, United States). Group differences were considered significant at *p* < 0.05. Principal component analysis (PCA) graphs were created using the R software (R Core Team, Vienna, Austria) with the *FactoMineR* and *factoextra* packages. Heatmaps were created using the Bioinfo Intelligent Cloud online tool (Chen et al., 2022).

## Results

### Identification and growth-promoting characteristics of isolate CNUC9

**Figure 1:**
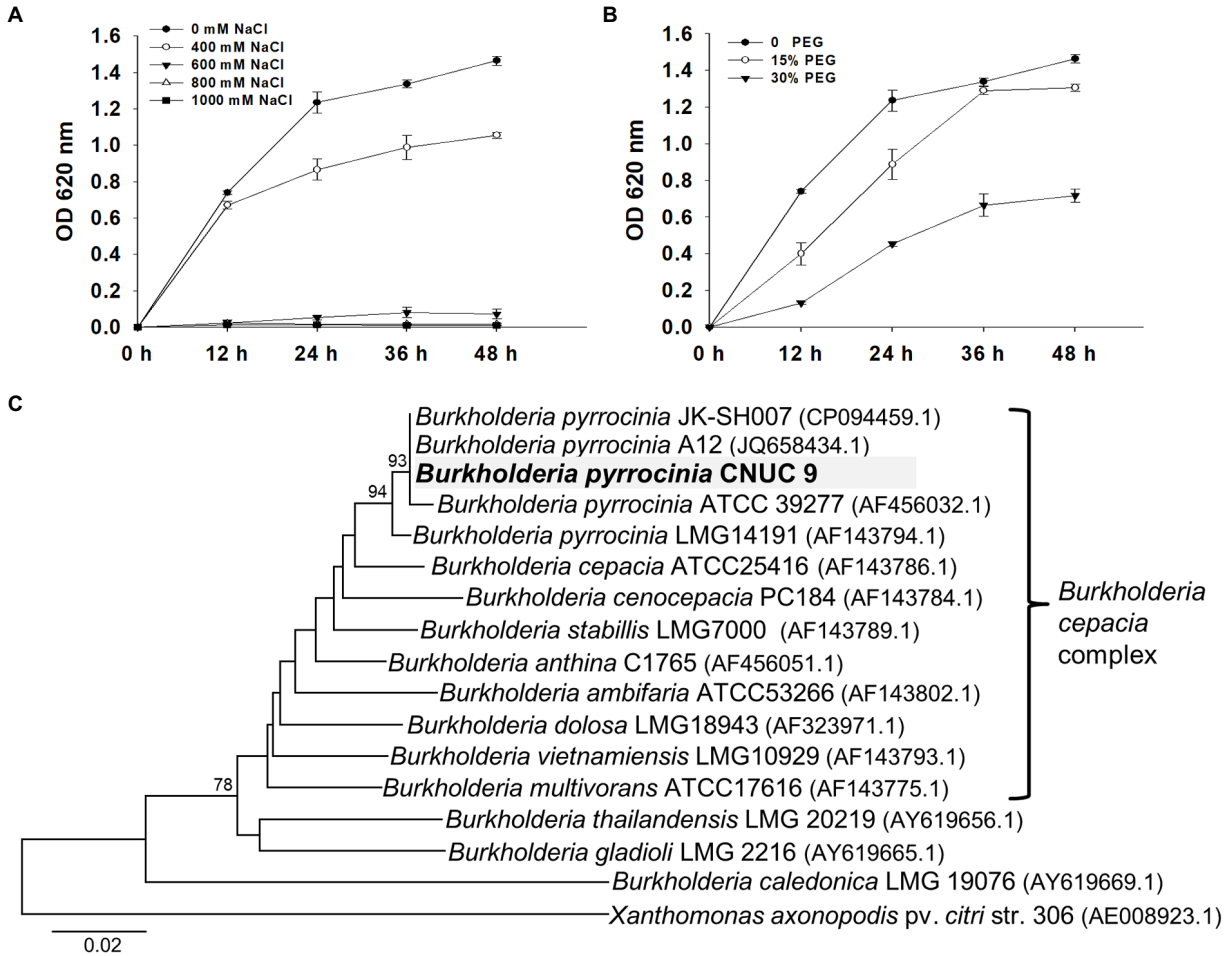
Growth curve and phylogenetic analysis of *B. pyrrocinia* CNUC9. Data for *B. pyrrocinia* CNUC9 growth curves were measured in liquid LB medium supplemented with **(A)** different NaCl concentrations (0, 400, 600, 800, or 1,000 mM), or **(B)** PEG 6000 concentrations (0, 15%, or 30%) incubated at 28°C for 48 h with rapid agitation. Error bars represent standard error (SE, *n* = 9). **(C)** Maximum likelihood tree inferred from analysis of *recA* gene sequences of *Burkholderia* species. Bootstrap values ≥70% based on 1,000 replicates are shown at the nodes. *Xanthomonas axonopodis* pv. *citri* str. 306 was used as an outgroup. Evolutionary distances were computed using the Kimura 2-parameter method and were expressed in numbers of base substitutions per site. Evolutionary analyses were conducted using the MEGA v6 software.

To discover bacteria that promote plant growth and salt tolerance, we isolated bacteria from the maize rhizosphere. Total of 24 isolates out of 139 isolates were selected according to their morphological characteristics and examined for plant growth-promoting traits. Among these bacteria, isolate CNUC9 showed the highest ACC deaminase production (292.5 nM). This isolate also produced IAA (9.8 μg/ml) and siderophore and exhibited substrate solubilization of casein and tricalcium phosphate (Supplementary Figure S1). These results suggest that CNUC9 has potential as a PGPR, providing nutrients to plants. CNUC9 also showed tolerance of up to 400 mM NaCl salt stress and 30% PEG6000 osmotic stress in LB medium (Figures 1A,B), indicating that it may perform efficiently in saline or drought environments.

Based on a BLASTN search of the 16S rRNA gene sequence, CNUC9 shared 100% homology with Bcc, including *B*. *cepacia*, *B. ambifaria*, and *B*. *pyrrocinia*. To further identify this strain, we performed *recA* gene sequence analysis. BLAST analysis indicated that the *recA* gene sequences of CNUC9 shared 100% identity with *B*. *pyrrocinia* strain JK-SH007 (accession no. CP094459). In a phylogenetic tree, CNUC9 clustered within the Bcc group and with its nearest neighbor as *B*. *pyrrocinia* A12 (JQ658434) and *B*. *pyrrocinia* JK-SH007, which has growth-promoting effects on tobacco seedlings (Han et al., 2012) and has biocontrol potential on polar canker (Ren et al., 2011), respectively (Figure 1C). Therefore, CNUC9 was identified as *B*. *pyrrocinia* based on 16S rRNA (accession no: ON076876) and *recA* (accession no: ON086316) gene sequences, which have multiple plant growth-promoting traits. Among bacterial determinants on plant growth promotion, bacterial VOCs displayed many advantages to apply crop plant under field compared to previous agrochemicals and chemical fertilizers (Fincheira et al., 2021).

### CNUC9 VOCs increase germination and survival rates and biomass under salt stress condition

**Figure 2:**
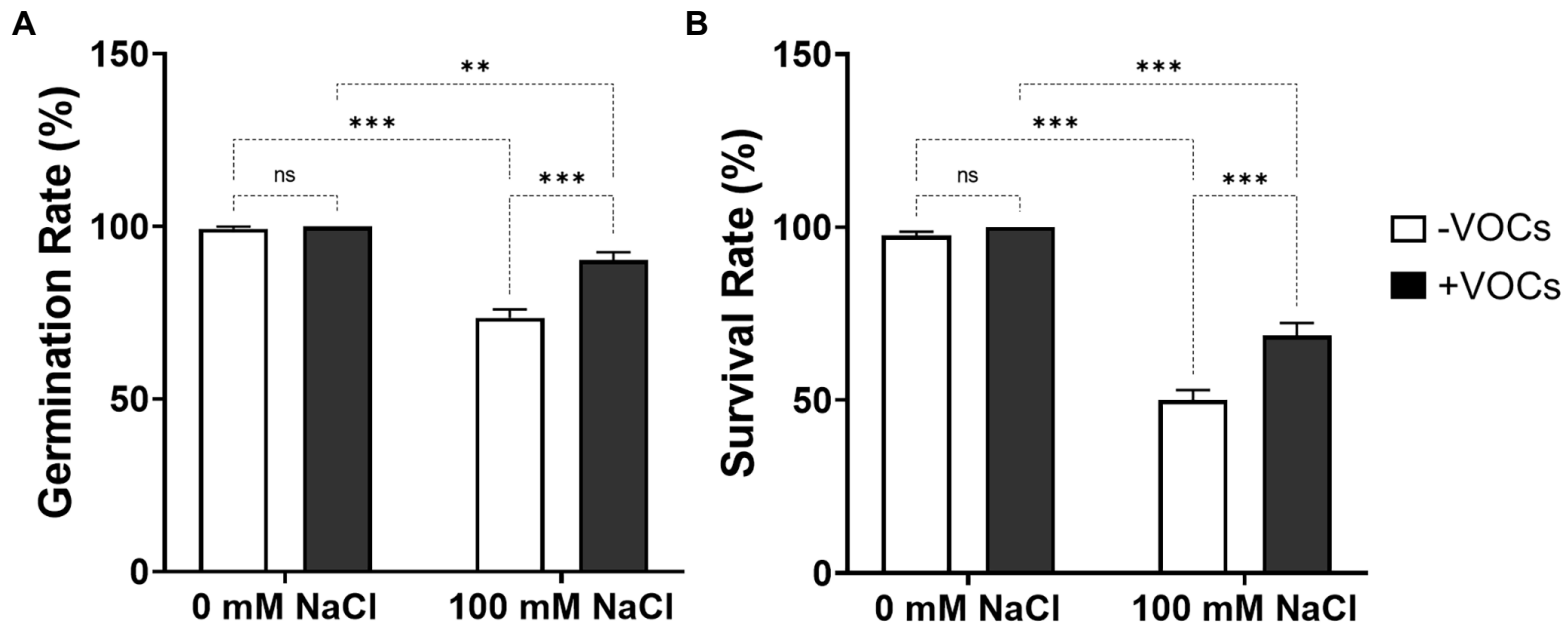
Effects of volatile organic compounds (VOCs) of *B. pyrrocinia* CNUC9 on *Arabidopsis* seed germination and survival rates under non-stress and salt stress conditions. **(A)** Germination rates and **(B)** survival rates were measured after 10  days. Error bars represent standard error of the mean (SEM) of three independent biological replicates (*n* = 200 seedlings per replicate). Asterisks on bars of the same parameter indicate statistical differences among treatments according to two-way analysis of variance (ANOVA), followed by Tukey’s multiple comparison test (**p ≤ 0.01; ***p ≤ 0.001; *n* = 600). ns, non significant.

To investigate the effects of VOCs emitted by CNUC9 on plant growth and abiotic stress tolerance, *A*. *thaliana* Col-0 seeds were sown on 1/2 MS medium with and without 100 mM salt and co-cultured with CNUC9 on I-plates (Figure 2). Under no salt stress (0 mM NaCl), there were no significant differences in *A*. *thaliana* seed germination or survival rates with or without bacterial VOCs exposure (*p* > 0.05). However, salt stress caused significant reductions in germination (25.9%) and survival rates (48.7%) among seedlings not exposed to VOCs, compared to control seedlings grown without salt stress. Interestingly, VOCs-exposed seedlings under salt stress had 22.6 and 37.3% higher germination and survival rates than non-exposed seedlings.

**Figure 3:**
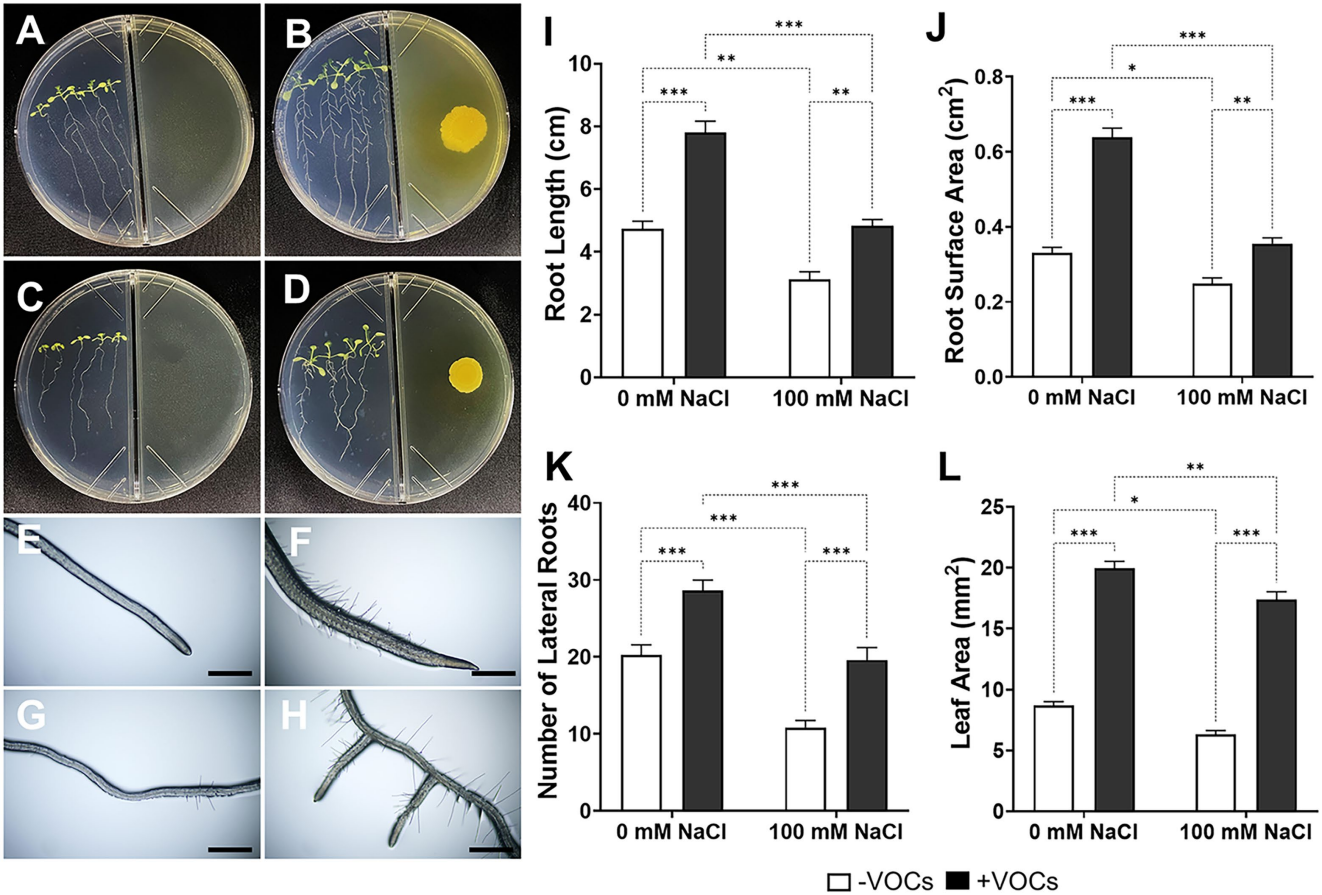
Effects of *B. pyrrocinia* CNUC9 VOCs on growth of *A. thaliana* seedlings under non-stress (0 mM NaCl) and salt stress (100 mM NaCl) conditions for 10  days. **(A–D)** CNUC9 VOCs promoted the growth of *A. thaliana* seedlings. **(A)** No VOCs exposure; 0 mM NaCl. **(B)** CNUC9 VOCs exposure; 0 mM NaCl. **(C)** No VOCs exposure; 100 mM NaCl. **(D)** CNUC9 VOCs exposure; 100 mM NaCl. **(E,F)** Root tips under salt stress under **(E)** no VOCs exposure or **(F)** CNUC9 VOCs exposure. **(G,H)** Lateral roots under salt stress under **(G)** no VOCs exposure or **(H)** CNUC9 VOCs exposure. **(I–L)** Quantitative analysis of *A. thaliana* biomass after 10  days of exposure to CNUC9 VOCs under no-stress and salt stress conditions. **(I)** Root length. **(J)** Root surface area. **(K)** Lateral root numbers. **(L)** Total leaf area. Error bars represent SEM of three independent biological replicates (*n* = 20 seedlings per replicate). Asterisks on bars of the same parameter indicate statistical differences among treatments (two-way ANOVA followed by Tukey’s test; *p ≤ 0.05; **p ≤ 0.01; ***p ≤ 0.001; *n* = 60). ns, non significant.

VOCs produced by CNUC9 triggered a number of physiological changes in *A*. *thaliana*. Following exposure to VOCs from CNUC9 in I-plate culture (0 or 100 mM NaCl) for 10 days, *A*. *thaliana* seedlings displayed increased biomass, in terms of lateral root numbers and extensive leaf area (Figures 3A–H). The growth parameters were further quantified using the WinRHIZO software (Figures 3I–L). Under non-stress conditions, VOCs-exposed seedlings showed significantly increased root length (64.7%), root surface area (93.0%), and lateral root numbers (41.3%) compared to non-exposed seedlings. Similar results were obtained for seedlings grown under salt stress. Seedlings treated with 100 mM NaCl that exposed to VOCs were exhibited significantly increased root length (54.9%), root surface area (42.8%), and lateral root numbers (80.9%) compared to non-exposed seedlings. These data demonstrate that VOCs from CNUC9 altered root architecture and enhanced root growth in *A*. *thaliana* seedlings with or without salt stress. Salt stress significantly reduced seedling root length, root surface area, and lateral root numbers, by 34.2, 24.9, and 46.5%, respectively, whereas VOCs-exposed seedlings showed similar root parameter levels to the controls without salt stress. Interestingly, *A*. *thaliana* seedlings exposed to VOCs had markedly larger leaf area (no salt stress, 129.6%; salt stress, 174.5%) than non-exposed control seedlings. Together, these results suggest that VOCs from CNUC9 greatly promoted plant growth and ameliorated salinity stress in *A*. *thaliana* seedlings by altering physicochemical properties in cellular solutes.

### CNUC9 VOCs increase leaf chlorophyll content

**Figure 4:**
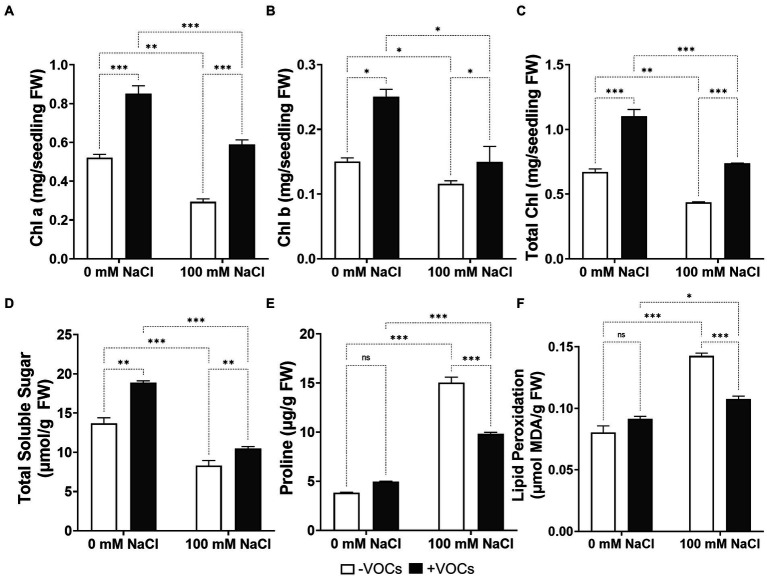
Effects of *B. pyrrocinia* CNUC9 VOCs on changes in **(A)** chlorophyll a, **(B)** chlorophyll b, **(C)** total chlorophyll, **(D)** total soluble sugar, **(E)** proline, and **(F)** malondialdehyde (MDA) content under no-stress (0 mM NaCl) and salt stress (100 mM NaCl) conditions for 10 days. Error bars represent SEM of three independent biological replicates (*n* = 20 seedlings per replicate). Asterisks on bars of the same parameters indicate statistical differences among treatments (two-way ANOVA followed by Tukey’s test; *p ≤ 0.05; **p ≤ 0.01; ***p ≤ 0.001; *n* = 60). ns, non significant.

To examine the impact of VOCs exposure on plant photosynthetic efficiency, the contents of leaf chlorophyll were measured. Under non-stress conditions, the levels of leaf Chl a, Chl b, and total chlorophyll were significantly increased in VOCs-exposed seedlings (*p* < 0.001), by 63.6, 66.4, and 64.2%, respectively, compared to non-exposed seedlings (Figures 4A–C). Similarly, seedlings exposed to VOCs for 10 days under salt stress conditions had 100.5, 29.0, and 69.0% higher leaf Chl a, Chl b, and total chlorophyll content, respectively, than non-exposed seedlings. Chl a, Chl b, and total chlorophyll content significantly decreased by 43.6, 23.0, and 34.9%, respectively, in non-exposed seedlings grown at 100 mM NaCl, whereas VOCs-exposed seedlings maintained leaf chlorophyll levels that were similar to those under non-stress conditions. These results indicate that exposure to VOCs from CNUC9 maintained photosynthetic pigments in *A*. *thaliana* seedlings, particularly under salt stress. These findings were consistent with the greater leaf area expansion as well as darker green leaves observed in *A*. *thaliana* seedlings exposed to CNUC9 VOCs than in non-exposed plants (Figure 3).

### CNUC9 VOCs modulate total soluble sugar, proline, and MDA content

Salt stress can damage the cellular membranes of plants and alter the production of osmoprotectants (Hasanuzzaman et al., 2013). To investigate whether CNUC9 VOCs affect physiological responses to osmoprotectants, endogenous levels of total soluble sugar, proline, and MDA were measured. The VOCs significantly enhanced the total soluble sugar content of seedlings compared to non-exposed seedlings grown under non-stress (38.0%) and salt stress (26.0%) conditions (*p* < 0.01; Figure 4D). Salt stress significantly reduced the total soluble sugar content by 39.3 and 44.6%, respectively, in non-exposed and VOCs-exposed *A*. *thaliana* seedlings.

Altered proline levels in plants are characteristic of salt stress. Plants without salt stress showed no significant difference in proline content with or without CNUC9 VOCs exposure (*p* > 0.05; Figure 4E). Under salt stress, proline content was dramatically increased by 293.7% in non-exposed seedlings compared to non-exposed seedlings without salt stress. The proline content of *A*. *thaliana* seedlings exposed to CNUC9 VOCs under salt stress increased by 97.78%, but was 34.8% lower than that of non-exposed seedlings.

Malondialdehyde (MDA) is one of the end products of lipid peroxidation and has been used as oxidative stress indicator in plant tissues during ROS damage (Gutteridge, 1995). Under salt stress, MDA content was significantly higher (77.6%) in non-exposed seedlings than in non-stressed control seedlings (*p* < 0.001; Figure 4F), whereas seedlings exposed to VOCs had 24.5% lower MDA content. Interestingly, seedlings under no salt stress exposed to CNUC9 VOCs had similar MDA content to non-exposed controls. Together, these results indicate that CNUC9 VOCs are involved in cellular membrane modulation and osmolyte protection in *A*. *thaliana* seedlings under salinity stress.

### CNUC9 VOCs modulate ROS accumulation

**Figure 5:**
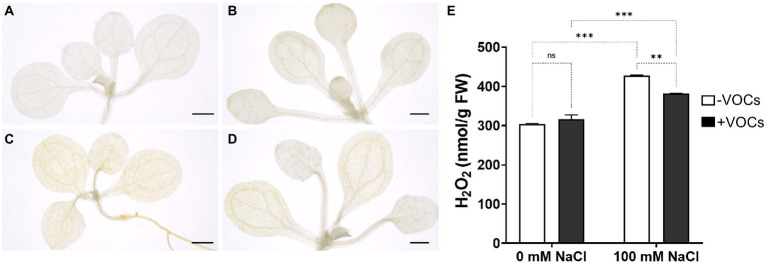
Effects of *B. pyrrocinia* CNUC9 VOCs on hydrogen peroxide (H_2_O_2_) modulation in *A. thaliana* seedlings. **(A–D)** *A. thaliana* leaves stained with 3,3′-diaminobenzidine (DAB) after seedling exposure to CNUC9 VOCs under no-stress and salt stress conditions for 10  days. **(A)** No VOCs exposure; 0 mM NaCl. **(B)** CNUC9 VOCs exposure; 0 mM NaCl. **(C)** No VOCs exposure; 100 mM NaCl. **(D)** CNUC9 VOCs exposure; 100 mM NaCl. Bar = 1 mm. **(E)** Quantification of H_2_O_2_ production in *A. thaliana* seedlings after exposure to CNUC9 VOCs under no-stress and salt stress conditions. Error bars represent SEM of three independent biological replicates (*n* = 20 seedlings per replicate). Asterisks on bars of the same parameters indicate statistical differences among treatments (two-way ANOVA followed by Tukey’s test; **p ≤ 0.01; ***p ≤ 0.001; *n* = 60). ns, non significant.

DAB polymerization was not observed in either VOCs-exposed or non-exposed seedlings under non-stress conditions, whereas extra brown precipitates indicating the presence of H_2_O_2_ were detected in non-exposed leaves under 100 mM NaCl stress (Figures 5A-C). Light brown precipitates were also observed in VOCs-exposed seedlings under 100 mM NaCl salt stress, indicating decreased H_2_O_2_ levels (Figure 5D). Interestingly, there was significantly less accumulation of brown precipitate in younger leaves than in older leaves. These results were further supported by an H_2_O_2_ accumulation assay. Under 100 mM NaCl stress, H_2_O_2_ levels were significantly higher (40.5%) in non-VOCs-exposed seedlings than in non-stressed control seedlings (*p* < 0.001), whereas in VOCs-exposed *A*. *thaliana* seedlings, H_2_O_2_ levels were significantly decreased by 10.7% compared to non-exposed seedlings (*p* < 0.01; Figure 5E). DAB staining and ROS accumulation assay results revealed that CNUC9 VOCs exposure reduced H_2_O_2_ accumulation in salt-stressed *A*. *thaliana* seedlings.

### CNUC9 VOC profiling

**Figure 6:**
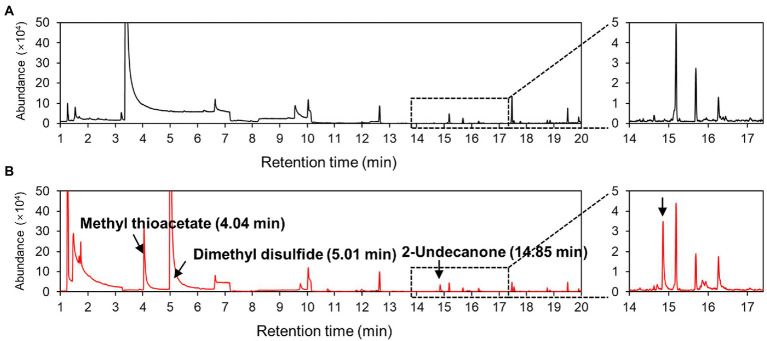
Gas chromatography–mass spectrometry (GC–MS) analysis of VOCs of *B. pyrrocinia* CNUC9 grown on nutrient agar (NA) medium for 3  days. **(A)** NA medium control. **(B)** *B. pyrrocinia* CNUC9.

To identify the VOCs emitted by CNUC9, we conducted SPME–GC–MS analysis on CNUC9 at 72 h post-inoculation (Figure 6). Three peaks were identified from CNUC9, among which the major peak areas were DMDS (*ca.* 87.71% of the total peak area; retention time [RT], 5.01 min) and methyl thioacetate (*ca.* 11.67%; RT, 4.04 min). The compound 2-undecanone (*ca.* 0.61%; RT, 14.85 min) was present in relatively low amounts; other detected compounds did not differ significantly from the uninoculated medium (control).

### Effects of pharmaceutical application of VOCs on plant growth

**Figure 7:**
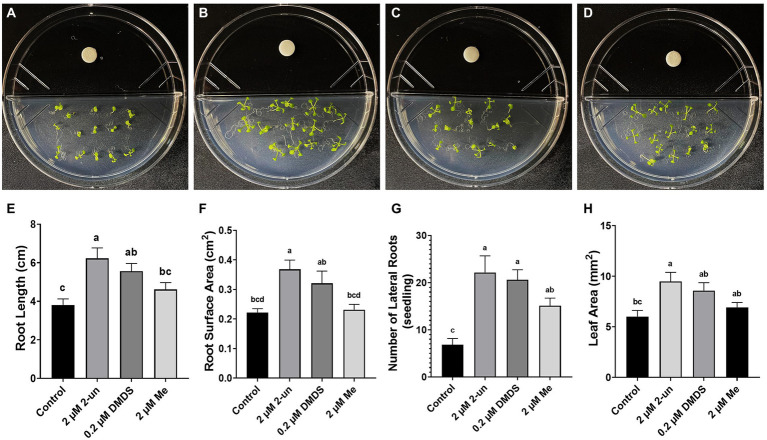
Effect of pure synthetic compounds on *A. thaliana* seedling growth after 10  days under no-stress conditions. Representative photographs of **(A)** control seedlings and seedlings treated with **(B)** 2  μM 2-undecanone, **(C)** 0.2  μM dimethyl disulfide, **(D)** 2  μM methyl thioacetate. **(E–H)** Growth parameters of representative *A. thaliana* seedlings including **(E)** root length, **(F)** root surface area, **(G)** lateral root numbers, and **(H)** total leaf area. Error bars represent SEM of three independent biological replicates (*n* = 20 seedlings per replicate). Different letters indicate significant differences between treatments (one-way ANOVA followed by Tukey’s test; *p* < 0.05).

To determine the effects of identified VOCs on plant growth, we tested three concentrations of DMDS, methyl thioacetate, and 2-undecanone using I-plates. We found that only 2-undecanone and DMDS promoted aerial and root growth in *A*. *thaliana* seedlings (Figure 7). Among the three concentrations, 2 μM 2-undecanone-exposed seedlings had increased root surface area (65.6%), root length (63.3%), lateral root numbers (222.6%), and leaf area (57.6%) compared to control groups (Figure 7; Supplementary Figure S2). In addition, seedlings exposed to 0.2 μM DMDS showed significantly increased root surface area (44.3%), root length (45.9%), lateral root numbers (200.2%), and leaf area (42.7%) compared to control groups; however, these levels were decreased compared to seedlings treated with 2 μM methyl thioacetate. Interestingly, higher VOCs concentrations had negative effects on seedling growth. Notably, significant growth inhibition occurred under treatment with 20 μM 2-undecanone, in terms of decreased root length (86.4%), root surface area (90.9%), lateral root numbers (59.2%), and leaf area (78.6%) compared to the control groups (Supplementary Figure S2). These results suggest that 2-undecanone and DMDS are major CNUC9 VOCs promoting seedling growth in a dose-dependent manner.

### Principal component analysis and heatmap analysis

**Figure 8:**
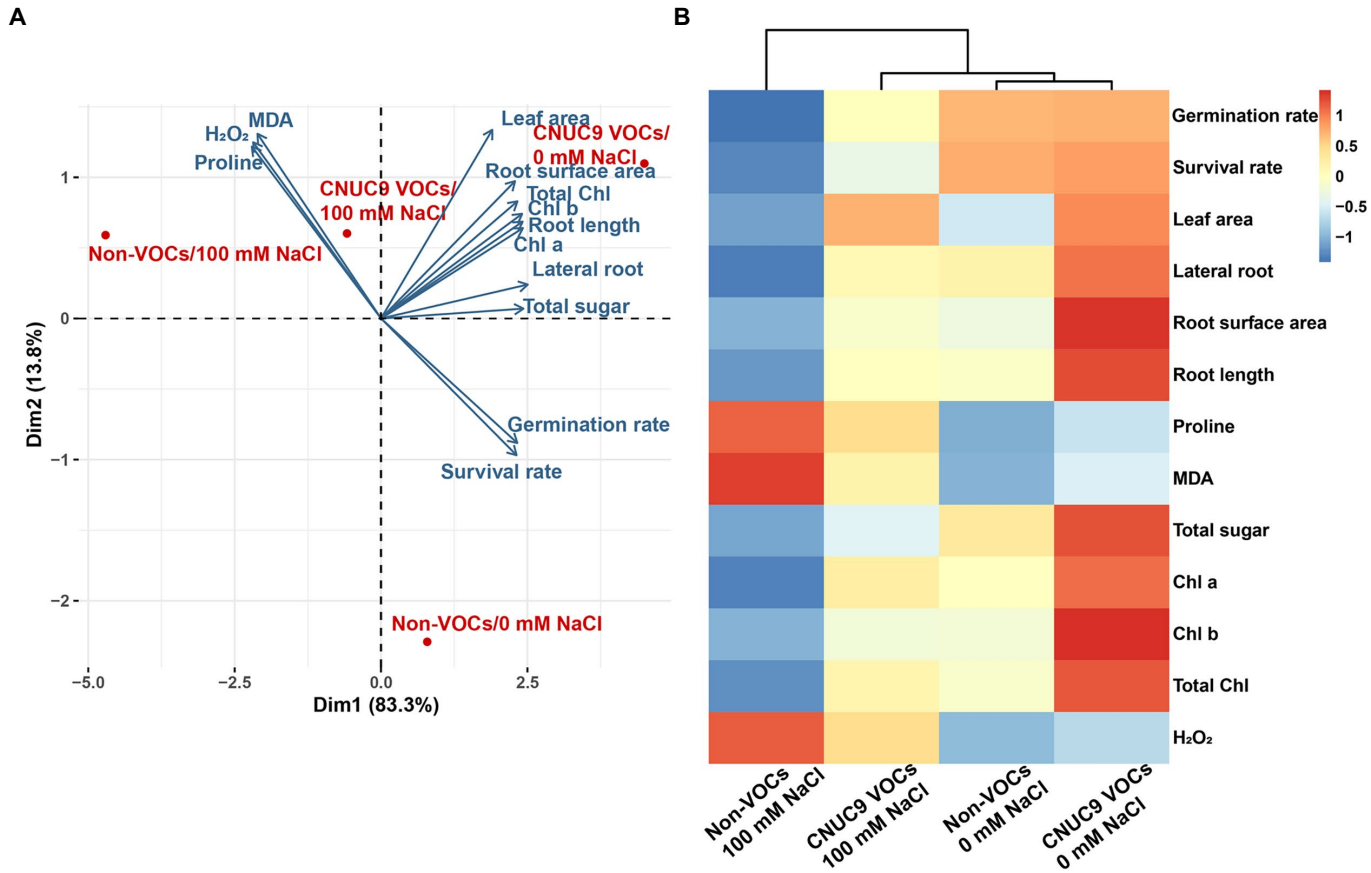
**(A)** Principal component analysis and **(B)** heat map responses of Pearson’s correlation coefficient for all growth variables of *A. thaliana* treated with *B. pyrrocinia* CNUC9 VOCs grown under salinity stress. Correlations are indicated in blue (positive) or red (negative).

We investigated possible relationships between different plant growth parameters and CNUC9 VOCs exposure effects under non-stress and salt stress conditions using Principal Component Analysis (PCA) based on the mean values of all variables. A bi-plot was inferred from the PCA-separated plant responses of the first two components, with overall 97.1% variability (PC1: 83.3%; PC2: 13.8%; Figure 8A). All treatments showed comparable morphological, biochemical, and physiological effects under CNUC9 VOCs exposure and salt stress. PCA results revealed that seedlings exposed to CNUC9 VOCs had significantly enhanced plant organ development (leaf area, root tips, and surface area), photosynthetic efficiency (Chl a, Chl b, and total chlorophyll content), and osmoprotectant accumulation (soluble sugar) compared to control inoculation under non-stress conditions. Similarly, CNUC9 VOCs treatment of stressed *A*. *thaliana* seedlings induced different H_2_O_2_, MDA, and proline content responses from the non-inoculation control.

Heatmap analysis showed that plant growth variables exhibited differential responses under different treatments (Figure 8B). Multivariate heatmap analysis suggested that salinity stress had positive effects on MDA, proline, and H_2_O_2_ accumulations in *A*. *thaliana* seedlings, but was negatively correlated with germination and survival rates, root proliferation, chlorophyll content, and sugar content in seedlings. By contrast, plants treated with CNUC9 VOCs positively influenced the vegetative, physiological, and photosynthetic pigments of *A*. *thaliana* seedlings under non-stress conditions, promoting plant growth. Heatmap results also indicated that CNUC9 VOCs maintained plant growth indices to non-stress levels under salt stress.

## Discussion

Present study, we newly isolated and characterized the PGPR strain *B. pyrrocinia* CNUC9 from maize rhizosphere. This isolate exhibited the ability to produce ACC deaminase, siderophore, protease, IAA, and able to solubilize calcium phosphate. Previously, endophytic *B*. *pyrrocinia* JK-SH007 was reported to promote plant growth and suppress poplar stem canker disease (Ren et al., 2011). *Burkholderia pyrrocinia* P10 has also been shown to significantly enhance peanut seedling growth under saline conditions (100 and 170 mmol/l NaCl) by secreting IAA, solubilizing phosphorus compounds, and producing siderophores and ACC deaminase (Han et al., 2021). Thus, *B*. *pyrrocinia* shows potential as a PGPR or biocontrol candidate *via* direct interaction with plants. However, our results clearly indicate that VOCs produced by *B*. *pyrrocinia* CNUC9 enhance salt tolerance in *A*. *thaliana* seedlings, showing significant increases in germination and survival rates, plant development, photosynthetic component regulation, antioxidant activity, and osmoprotectant responses in the absence of any direct contact with plants.

Recent studies have demonstrated that VOCs emitted by PGPR have the ability to improve plant growth and biomass without physical contact, potentially enhancing plant resistance against biotic and abiotic stresses (Ryu et al., 2003; Park et al., 2015; Raza et al., 2016; Cordovez et al., 2018; Liu et al., 2020a). We also observed that *A. thaliana* seedlings exposed to *B. pyrrocinia* CNUC9 VOCs showed significantly enhanced growth compared to non-VOCs-exposed plants under both normal and salt stress conditions. As shown in Figure 3, representative *A. thaliana* seedlings treated with CNUC9 VOCs reached the 5-leaf stage (non-stress) and 4-leaf stage (salt stress) after 10 days of co-cultivation, whereas control plantlets had 4 leaves (non-stress) and 3 leaves (salt stress). VOCs-exposed *A. thaliana* seedlings also showed 2.3-fold (non-stress) and 2.7-fold (salt-stress) increases in total leaf area compared to non-exposed controls. Similarly, Tahir et al. (2017) and Zhang et al. (2007) reported that bacterial volatiles had a significant increase leaf area and leaf expansion by modulating expressions of cell division and auxin related genes.

The root system is responsible for water and nutrient acquisition from the soil; thus, a larger root system architecture increases plant resilience and survival under various stresses. Root system architecture is coordinated by plant growth hormones including auxin and cytokinin (Wang et al., 2021; Rivas et al., 2022); previous studies reported that beneficial soil microbes directly regulate the accumulation of these hormones in plant roots (Wu et al., 2012; Wang et al., 2021). Interestingly, volatiles released by PGPR also significantly improve root architecture through modulating the root hormonal networks that contribute to water and nutrient uptake capacity (Wang et al., 2006; Sharifi et al., 2021). We observed adverse effects of salt stress on *A. thaliana* root system architecture in terms of significantly reduced lateral root numbers and root hair development, as well as decreased root length and surface area (Figure 3). However, exposure to CNUC9 VOCs markedly alleviated the negative effects of salt stress, and all root development parameters recovered to levels similar to the control group under no salt stress (Figure 3). Recently, Li et al. (2021) demonstrated that VOCs produced by *Bacillus* species participated in regulating lateral root development in *A. thaliana* seedlings *via* an auxin-dependent mechanism. By contrast, *B. subtilis* GB03 volatiles triggered growth promotion through cytokinin–ethylene signaling, whereas *B. amyloliquefaciens* IN937a volatiles appeared to act independently of both cytokinin and ethylene (Ryu et al., 2003; Farag et al., 2006). These findings suggest that different types of volatile compounds emitted by PGPR may differentially regulate plant hormones *via* different signaling pathways. We demonstrated that VOCs produced by CNUC9 significantly promoted root system architecture formation in *A. thaliana* seedlings; the specific mechanisms of VOCs-mediated phytohormone responses are currently being investigated.

Under salt stress, plants experience physiological and biochemical changes that lead to the accumulation of an array of metabolites such as chlorophyll contents, total soluble sugar, and proline in cells (Kumar et al., 2017). PGPR stimulates plants to activate different physiological and biochemical mechanisms to cope with stress by accumulating osmotic regulator solutes to maintain osmotic pressure homeostasis and structural stability (Acosta-Motos et al., 2017). Chlorophyll is a green pigment that has a vital role in photosynthesis. Maintaining endogenous chlorophyll levels is important for photosynthetic efficiency to acquire energy for growth and development. Under salt stress, however, chloroplast enzyme activity is increased, followed by the acceleration of chlorophyll degradation and decreased photosynthesis efficiency (Megdiche et al., 2008). Our results showed that salt stress adversely affected Chl a, Chl b, and total chlorophyll content; however, CNUC9 VOCs exposure significantly increased chlorophyll content compared to non-exposed controls under non-salt-stress conditions and successfully protected photosynthetic pigment levels under 100 mM salt stress (Figures 4A–C). Our results are consistent with those of a previous report that exposure to *B*. *subtilis* GBO3 VOCs stimulated photosynthetic activity by increasing chlorophyll content and upregulating chloroplast gene expression (Zhang et al., 2008). Additionally, VOCs of *B*. *subtilis* SYST2 and *B*. *amyloliquefaciens* FZB42 increase chlorophyll synthesis, which helps mitigate the negative effects of saline stress on photosynthesis and enhancing plant growth (Tahir et al., 2017).

Total soluble sugar and proline are often used as indicators of potential stress reactions in plants. Total soluble sugar is a main product of photosynthesis and a fundamental component of energy supply to cells for carbohydrate metabolism. However, in plants under stress, soluble sugar acts as a major osmoregulation substance (Zang et al., 2019). Proline is another metabolite that acts as an osmoprotectant and antioxidant defense molecule (e.g., scavenging hydroxyl free radicals) that helps maintain osmotic balance and lower ROS concentrations under stress (Kumar et al., 2017). The application of PGPR is beneficial for total sugar and proline accumulation in plants against osmotic stress caused by salinity (Abbas et al., 2019). For example, inoculation of *B*. *subtilis* SU47 and *Arthrobacter* sp. SU18 enhanced the total soluble sugar and proline content of wheat under salt stress compared to non-inoculated control plants (Upadhyay et al., 2012). The PGPR strain *Kocuria rhizophila* Y1 also increased soluble sugar and proline content in maize under salt stress (Li et al., 2020a). Similar results were obtained in the current study, as total sugar content increased significantly in plants under CNUC9 VOCs exposure and either non-stress or salt stress conditions (Figure 4D). By contrast, proline content was significantly lower in plants exposed to VOCs than in non-exposed plants under salt stress (Figure 4E). This finding is consistent with previous studies that have reported that PGPR treatment decreases proline content but increases total sugar content (Hmaeid et al., 2019; Li et al., 2020a; Liu et al., 2020b). Such studies suggested that proline accumulation is lower in the presence of PGPR because plants treated with PGPR do not experience high salt stress.

ROS play important roles as signaling molecules in the regulation of plant adaptive defense responses against biotic and abiotic stresses (Kumar et al., 2017). Under stress, plants overproduce ROS, resulting in chlorophyll degradation, cell membrane damage through lipid peroxidation, and electrolyte leakage (Kumar and Singh, 2016). Lipid peroxidation is a ROS-mediated cellular damage reaction that targets polyunsaturated fatty acids in the cell membrane and generates MDA as a final metabolite (Kumar et al., 2017; Alexander et al., 2020). Thus, MDA content reflects ROS production in plant tissues during stress and is responsible for cellular membrane instability (Gutteridge, 1995). Under salt stress, we observed marked reductions in H_2_O_2_ and MDA concentrations in CNUC9 VOCs-exposed *A*. *thaliana* seedlings compared to non-exposed seedlings (Figures 4, 5). Those results suggest that CNUC9 VOCs alleviated oxidative damage in plants due to salt stress and boost the membrane stability.

We detected three VOCs produced by *B*. *pyrrocinia* CNUC9; among these, we concluded that DMDS was the major VOC influencing stress responses in *A*. *thaliana* in this study, which is consistent with the findings of a previous study on strain *B*. *pyrrocinia* JK-SH007 (Liu et al., 2020a). DMDS is common to most of bacterial species, e.g., *Pseudomonas*, *Serratia*, *Bacillus*, and *Stenotrophomonas*, which show antifungal activity, influence mosquito behavior, enhance plant growth, and reduce potential fungal toxin production (Meldau et al., 2012; Popova et al., 2014; Tyagi et al., 2019). DMDS also accelerated the growth of tobacco plants by increasing sulfur content in the environment (Meldau et al., 2013), and induced an auxin response in lateral root primordia in *A*. *thaliana* Tyagi et al. (2019). However, Cordovez et al. (2018) reported that DMDS had no impact on shoot growth in *A*. *thaliana* and only a slight effect on root growth. In our study, the plant-promotion efficiency of DMDS was concentration-dependent. The 2-undecanone belongs to medium-chain methyl ketones family which exhibit low water solubility and high volatility (Yan et al., 2020). This volatile is a major VOC of *Pseudomonas* spp. and *Bacillus* spp. (Gu et al., 2007; Timm et al., 2018), which can stimulate seed germination in *Lactuca sativa* (Fincheira et al., 2017) and display nematocidal and antifungal activities (Gu et al., 2007; Li et al., 2020b). We also found that an optimal dose of 2-undecanone had strong growth-promotion ability, whereas higher concentrations led to growth inhibition (Supplementary Figure S2).

In addition to those VOCs, bacterial inorganic volatiles are also reported to affect plant growth: ammonia and hydrogen cyanide are considered harmful (Blom et al., 2011b; Weise et al., 2013), while CO_2_, nitric oxide and hydrogen sulfide are reported beneficial to plant growth and abiotic stress tolerance (Kai and Piechulla, 2009; Christou et al., 2013; Sharma et al., 2021). Therefore, in the sealed Petri dish, the accumulation of the former volatiles can induce growth inhibition whereas accumulation of the latter can promote plant growth. For example, as an essential substrate for photosynthesis, CO_2_ has been suggested as a plant growth promoting compound. Kai and Piechulla (2009) demonstrated that the growth promoting effect of *Serratia odorifera* 4Rx13 was specific to the sealed Petri dish environment, not to the open cultivations, indicating the enhanced effect of bacteria-produced CO_2_ on plant growth. Zhang et al. (2021) compared the plant growth in the tightly sealed (high CO_2_) or open (ambient CO_2_) systems and elucidated the role of CO_2_ as a key contributor to the plant growth-promoting volatiles emitted by bacteria in a sealed system. However, others provided evidence for a lack of role for CO_2_ in plant growth promotion. Lee et al. (2012) presented that co-cultured PGPR strains induced a significantly enhanced growth promotion of *A. thaliana* by growing plants in the presence of Ba(OH)_2_, a chemical eliminator of CO_2_. Ledger et al. (2016) also assessed the effects of *Paraburkholderia phytofirmans* PsJN on plant growth in sealed-and non-sealed systems, and clarified that plant growth promotion was predominated by volatile-mediated effects, not by CO_2_ produced by PsJN. We showed that optimal doses of synthetic VOCs (DMDS and 2-undecanone) significantly promoted *A. thaliana* growth in the sealed Petri dish (Figure 7). However, we also noticed that the effects of growth promotion and salt stress tolerance were much greater when *A. thaliana* was co-cultured with CNUC9 compared with synthetic compounds (Figure 3; Supplementary Figure S2). These results suggest that plant growth promotion and salt stress tolerance may be induced in part by other volatile compounds such as CO_2_, nitric oxide and hydrogen sulfide produced by CNUC9 in addition to DMDS and 2-undecanone. Although further studies are still required, these data support the important roles of DMDS and 2-undecanone in plant growth promotion and resistance to salt stress.

Volatile mixtures depend strongly on the growth medium (Rath et al., 2018) and vary considerably among closely related species (Nawrath et al., 2012), among individuals of the same species from different origins (Groenhagen et al., 2013), and among inoculation doses (Blom et al., 2011a). Benzothiazole, dimethylthiomethane, and 11 other VOCs have been detected from *B*. *pyrrocinia* JK-SH007 cultured on LB medium. These compounds (excluding DMDS) were not found in our strain *B*. *pyrrocinia* CNUC9 grown on NA medium, indicating that VOCs production is highly cultivation medium-dependent. Blom et al. (2011a) showed that VOCs of *B*. *pyrrocinia* Bcc171 grown on Methyl Red and Voges–Proskauer (MR-VP) medium show greater growth promotion in *A*. *thialiana*, whereas VOCs of *B*. *pyrrocinia* Bcc171 grown on LB medium inhibit *A*. *thialiana* growth. Although in that study both strains were cultured on LB medium, strain *B*. *pyrrocinia* Bcc171 isolated from soil in the United States produced trans-2-dodecenal, 2-nonanone, 2-decanone, 2-undecanone, undecanal, tetrahydro-3-furanmethanol, 1-butoxy-2-propanol, phenol, and 3-methyl-1-butanol, none of which were detected on strain *B*. *pyrrocinia* JK-SH007 from poplar stems in China, which corresponds with results of Groenhagen et al. (2013). To date, VOCs biosynthesis pathway data are insufficient to understand these phenomena; further in-depth study of the metabolic processes involved in VOC biosynthesis and genetic regulation are required, and the mechanism of their action on various biological substances must be investigated.

## Conclusion

Our findings have shown that co-culture with *B*. *pyrrocinia* CNUC9 VOCs stimulated the development of the root system architecture, leaf proliferation, and induction of salt tolerance in *A*. *thaliana* seedlings. Based on VOC profiling analysis using SPME–GC–MS, we found that CNUC9 emitted three VOCs under our experimental conditions: DMDS, methyl thioacetate, and 2-undecanone. Among these three VOCs, optimal concentrations of dimethyl disulfide and 2-undecanone promoted *A*. *thaliana* growth and alleviated salt stress. Our findings provide a potential insight on the agricultural application of VOCs for salt-stress tolerance. Further study is required to elucidate the molecular mechanisms of VOCs-mediated systemic salt tolerance in plants.

## Data availability statement

The datasets presented in this study can be found in online repositories. The names of the repository/repositories and accession number(s) can be found in the article/Supplementary material.

## Author contributions

JMY: conceptualization, funding acquisition and project administration, review and editing. HL: investigation and experiments, data analysis, original draft preparation. C-MR and MR conducted GC–MS analysis. All authors have read and agreed to the published version of the manuscript.

## Funding

This work has supported by the National Research Foundation of Korea (NRF) grant funded by the Korea government (MSIT) (2020R1C1C1012005).

## Conflict of interest

The authors declare that the research was conducted in the absence of any commercial or financial relationships that could be construed as a potential conflict of interest.

## Publisher’s note

All claims expressed in this article are solely those of the authors and do not necessarily represent those of their affiliated organizations, or those of the publisher, the editors and the reviewers. Any product that may be evaluated in this article, or claim that may be made by its manufacturer, is not guaranteed or endorsed by the publisher.
