# Supplementary figures and images for: Volatile organic compounds emitted by Burkholderia pyrrocinia CNUC9 trigger induced systemic salt tolerance in Arabidopsis thaliana
Source: Front Microbiol. 2022 Nov 17;13:1050901. doi: 10.3389/fmicb.2022.1050901 (PMC9713481; doi:10.3389/fmicb.2022.1050901)

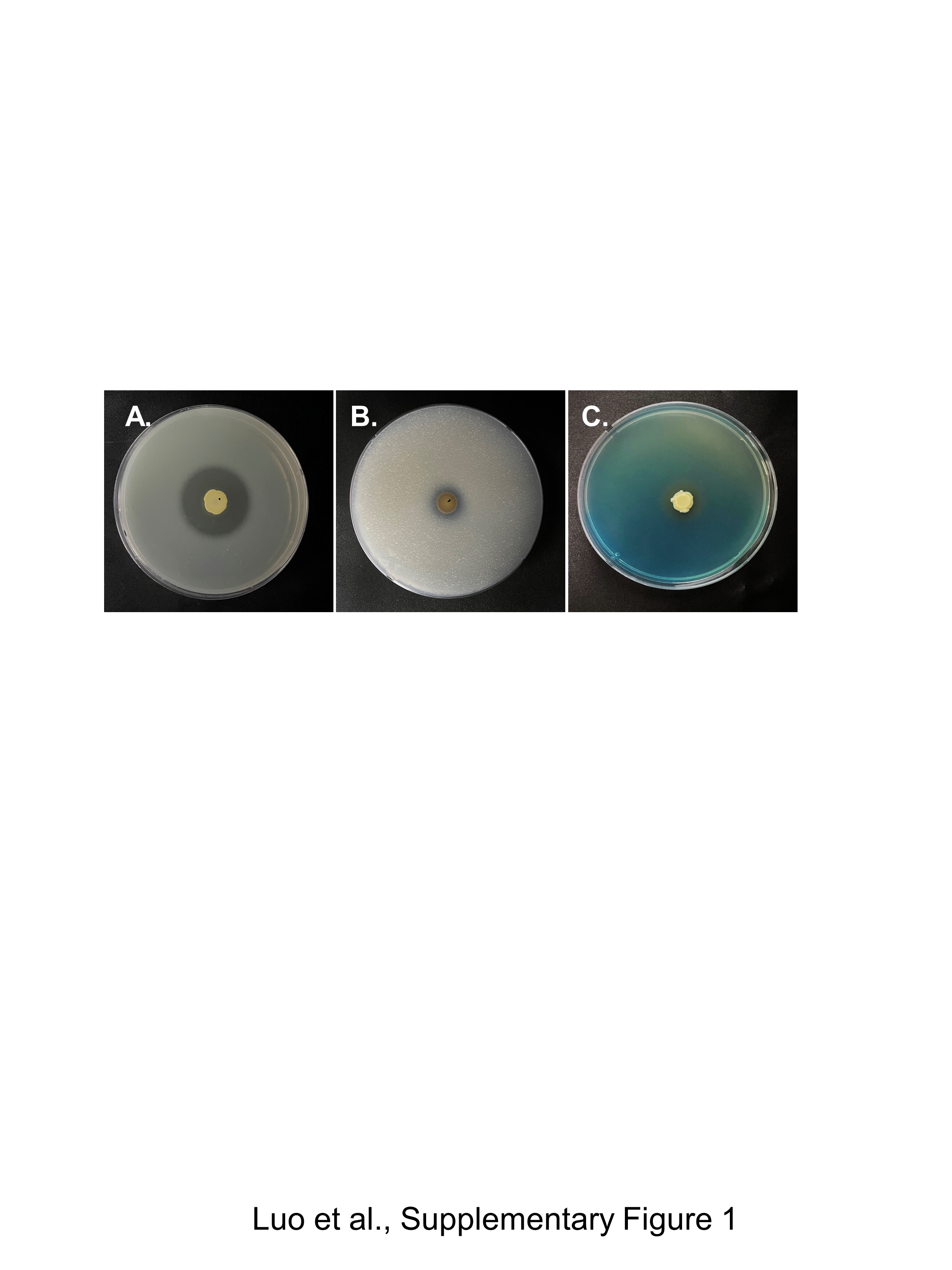

Supplement: Supplementary file 1 [file Image_1.tif]

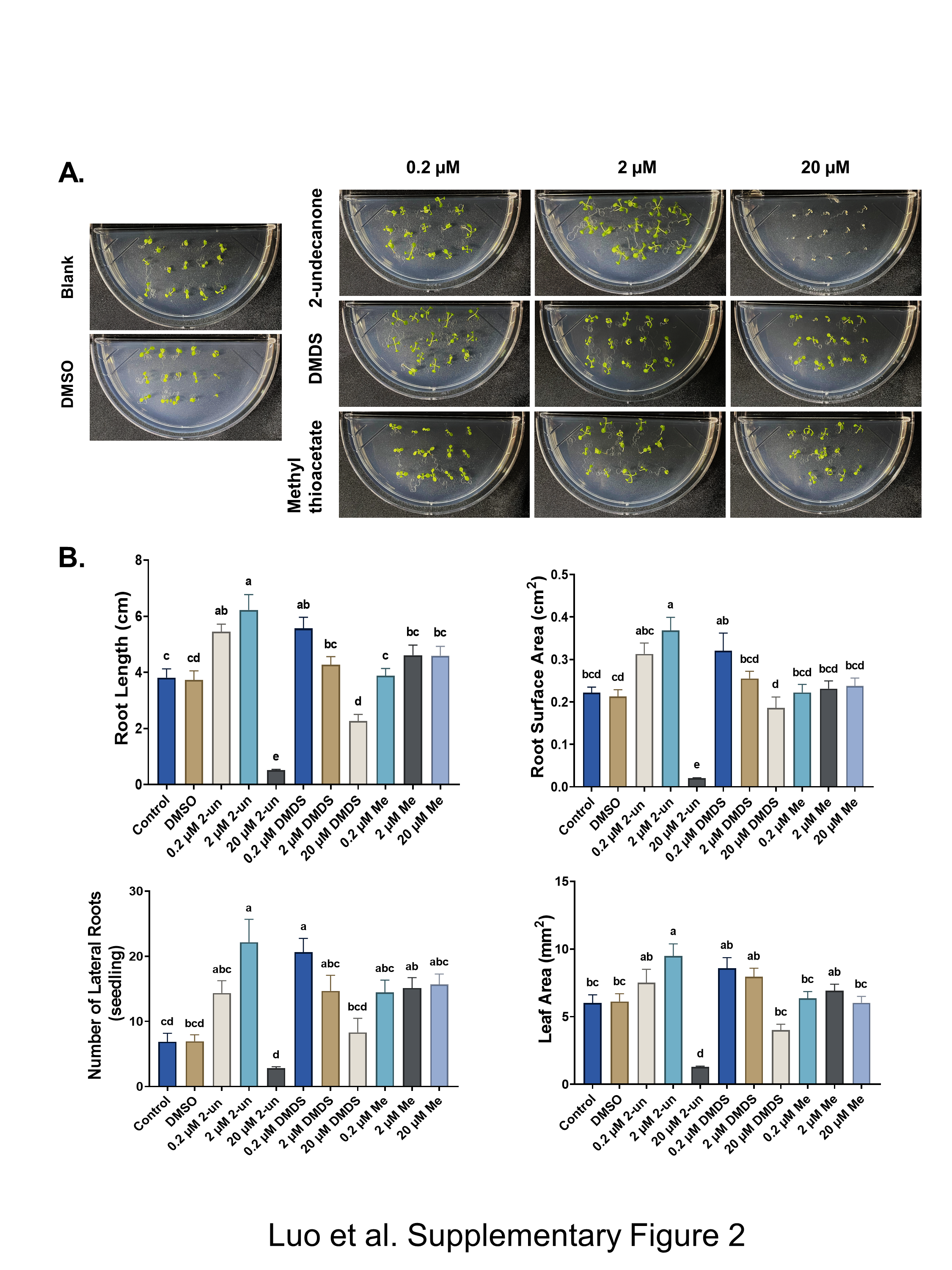

Supplement: Supplementary file 2 [file Image_2.tif]
